# Lycopene Alleviates T-2 Toxin-Induced Testosterone Synthesis Disorder by Inhibiting ROS/p38/TORC2/CREB Axis

**DOI:** 10.3390/antiox15091060

**Published:** 2026-08-25

**Authors:** Xu Yang, Hui Tang, Fengjuan Chen, Zeao Hua, Binbin Luo, Yanfei Li, Gongyi Chen, Cong Zhang

**Affiliations:** 1 College of Veterinary Medicine, Henan Agricultural University, Zhengzhou 450002, China; yangxu@henau.edu.cn (X.Y.); 19503883852@163.com (H.T.); fengjuanchen@stu.henau.edu.cn (F.C.); huazeao@stu.henau.edu.cn (Z.H.); 15082918757@163.com (B.L.); 2 College of Veterinary Medicine, Northeast Agricultural University, Harbin 150030, China; liyanfei@neau.edu.cn; 3 Pet Science College, Henan Vocational College of Agriculture, Zhengzhou 450002, China; chengongyi2008@163.com

**Keywords:** T-2 toxin, lycopene, ROS/p38/TORC2/CREB axis

## Abstract

T-2 toxin is a typical fusarium mycotoxin that induces reproductive injury. As a male reproductive toxicant, impaired testosterone biosynthesis is the primary reproductive toxic effect induced by T-2 toxin. Therefore, clarifying how T-2 toxin disrupts testosterone synthesis at the molecular level and screening natural plant extracts that can alleviate this reproductive toxicity are of great importance. Lycopene (LYC) is a natural carotenoid known for its reproductive protective properties. Hence, this work assessed the protective efficacy and molecular mechanisms of LYC against T-2 toxin-caused testosterone synthesis disruption. Our results showed that LYC supplementation significantly alleviated testicular structural injury, restored testicular organ coefficients, and rescued serum and intracellular testosterone levels via transcriptional and translational upregulation of key steroidogenic proteins. Mechanistically, LYC suppressed T-2 toxin-triggered reactive oxygen species (ROS) accumulation, inhibited MAPK pathway activation, and prevented ROS/p38/TORC2/CREB axis activation, thereby restoring TORC2/CREB transcriptional activity and steroidogenesis. Pharmacological validation using SB203580 and NAC confirmed the central role of the ROS/p38/TORC2/CREB axis. Taken together, LYC alleviates T-2 toxin-triggered disruption of testosterone synthesis by inhibiting the ROS/p38/TORC2/CREB axis, and we identify LYC as a promising nutritional strategy against mycotoxin-caused reproductive toxicity.

## 1. Introduction

T-2 toxin, among the most hazardous type A trichothecenes, can be frequently detected in many food commodities, such as nuts, cereals, herbs, and fruits. Testing 233 nut samples from China revealed 47.6% of them contained T-2 toxin [1]. In 120 grain samples (maize, barley, wheat and rice) from Algeria, T-2 toxin was detected in 50% [2]. Crops and food are susceptible to T-2 toxin contamination at various stages of cultivation, harvesting, processing, and storage. Therefore, the World Health Organization considers T-2 toxin to be a significant source of dietary contamination and a major health risk to humans and animals [3,4,5]. In both humans and animals, chronic or prolonged exposure to a T-2 toxin-tainted diet can lead to harm to multiple organ systems, including the brain, gastrointestinal tract, and hematopoietic and lymphoid tissue [6,7,8]. Among the numerous adverse impacts caused by T-2 toxin, reproductive organ damage is particularly significant. The male reproductive adverse effects of T-2 toxin have garnered significant attention recently owing to growing public awareness of environmental issues [9,10,11].

Testosterone is an androgen produced by Leydig cells and is essential for male gonadal development, spermatogenesis, and maintaining sexual behavior [12]. T-2 toxin-induced reproductive disruption has been consistently shown to correlate strongly with impairment of testosterone synthesis across numerous studies [12,13,14,15,16]. Initial proof linking T-2 toxin-caused impairment of male reproductive function to disruption of testosterone synthesis was reported in 1990 [12]. Since then, this perspective has been consistently supported by a substantial body of animal and cell experimental evidence [14,15,16]. Mouse experiments have shown T-2 toxin causes injury to the structural integrity in the male testes and the reproductive endocrine axis, reduces testosterone levels, and impairs male mating and the fertility index [14]. The synthesis of testosterone is intimately linked to the alteration of testosterone synthase. Our earlier work showed T-2 toxin suppresses testosterone production by downregulating testosterone synthase levels in mouse testes and TM3 cells [15]. Furthermore, in cellular models, T-2 toxin inhibited testosterone production in porcine Leydig cells, which is associated with decreased StAR and 3β-HSD protein levels [16]. Considering the aforementioned research, alleviating testosterone synthesis dysfunction may be the key treatment strategy against T-2 toxin-caused reproductive toxicity.

The MAPK pathway contains c-Jun N-terminal kinase (JNK) and extracellular-regulated protein kinase (ERK), along with p38 mitogen-activated protein kinase (p38). Evidence suggests that some endogenous/exogenous substances could regulate testosterone synthesis via the MAPK pathway [17]. Polystyrene nanoplastics inhibit StAR expression through triggering the AKT and ERK1/2 MAPK axis in mouse testicular tissue [18]. Oxidized LDL activates the p38 MAPK/COX-2 pathway to suppress the expression of testosterone synthesis enzymes, resulting in a decrease in testosterone synthesis in Leydig cells [19]. Our previous work identified that overactivation of MAPK signaling serves as the primary process behind T-2 toxin-caused impairment in testosterone synthesis [15]. CREB (cAMP response element-binding protein) is essential for regulating testosterone synthesis [5]. Transducers of regulated CREB2 (TORC2) mainly binds with p-CREB in the nucleus to form protein complexes which can bind on the promoter region of StAR protein, promoting StAR transcription and initiating testosterone synthesis [20]. When the TORC2 is phosphorylated, p-TORC2 dissociates from p-CREB and translocates to the cytoplasm, resulting in reduced transcriptional activity of StAR. Previous studies have shown that p38 can promote TORC2 phosphorylation, thereby inhibiting StAR transcription [21,22,23]. However, whether TORC2 contributes to T-2 toxin-caused disruption of testosterone synthesis via p38 activation has not yet been clarified. Therefore, dissecting the p38/TORC2/CREB axis in T-2 toxin-triggered testosterone synthesis disorder may offer novel therapeutic targets for alleviating its reproductive toxicity.

Lycopene (LYC) is a lipid-soluble red carotenoid tetraterpene compound and has powerful antioxidant effects [24,25]. LYC is predominantly present in vegetables and fruits that are red, pink, or orange in color, such as tomatoes, apricots, watermelon, red carrots, and guavas [26]. LYC has been shown to be effective in addressing reproductive problems, cancer, cardiovascular diseases, inflammatory conditions, and nerve diseases [27]. LYC cannot be synthesized in the body; instead, it must be ingested through food or dietary supplements [28]. Ingested LYC mainly accumulates in the testes, liver, prostate and adrenal gland, and the concentration of LYC in the testis is 10 times greater than that in other organs [29,30]. In varicocele rats, LYC can reverse the decreases in StAR protein expression and testosterone levels [31]. LYC alleviated the decrease in testosterone synthase protein expression (CYP17A1, 17β-HSD, 3β-HSD, StAR and CYP11A1) in TM3 cells exposed to di-n-butyl phthalate (DBP) and inhibited the testosterone synthesis disorder induced by DBP [32]. LYC can prevent the reduction in testosterone levels caused by zearalenone (ZEA) in male mice and protect the testis from reproductive damage caused by ZEA [33]. In addition, LYC can alleviate the decrease in testosterone expression in male rat testes caused by dimethoate, increase the number of spermatogenic cells and reduce oxidative stress, thus protecting the reproductive function of male rats [34]. These studies show that LYC can participate in testosterone production by regulating the expression of testosterone synthase. Furthermore, LYC was found to suppress MAPK signaling activation in a tramadol-induced hepatotoxicity model, an effect that may be attributed to its ability to alleviate lipid peroxidation and strengthen antioxidant defense mechanisms [35]. In a Sprague Dawley mouse model of ischemia, nanoliposomes derived from LYC were able to suppress apoptosis by inhibiting MAPK-JNK activation [36]. However, whether LYC ameliorates T-2 toxin-triggered male reproductive impairment through the p38/TORC2/CREB axis has yet to be established.

This work assessed whether LYC could relieve T-2 toxin-caused testosterone synthesis disorders through modulation of the p38/TORC2/CREB axis in vivo and in vitro, thereby providing a pharmacological reference for the management of toxin-related reproductive impairment.

## 2. Materials and Methods

### 2.1. Establishment of the Animal Model

In this investigation, one hundred and sixty 8-week-old male mice (C57BL/6J) were used as research objects and randomly divided into eight groups. All animals were bred as well as maintained at the Experimental Animal Center of Henan Agricultural University. All animal experiments were approved by the Institutional Animal Care and Use Committee of Henan Agricultural University (HNND2021030801). Housing conditions were kept at 22–24 °C with a 12 h light/dark cycle and 50–60% relative humidity. Animals were provided with a basal diet and water ad libitum throughout the study. There were four groups for the T-2 toxin poisoning model (*n* = 20): control group: 0 mg/kg body weight (BW) T-2 toxin (CG), Low-dose group: 0.5 mg/kg BW T-2 toxin (LG), Medium-dose group: 1 mg/kg BW (MG) and High-dose group: 2 mg/kg BW (HG) T-2 toxin (Pribolab, Qingdao, China). T-2 toxin was administered via oral gavage once daily for a total of 28 consecutive days. For the LYC intervention model (*n* = 20), Con group: control solvent; T-2 group: 1 mg/kg BW T-2 toxin; T-2 + LYC group: 1 mg/kg BW T-2 toxin + 5 mg/kg BW LYC; LYC group: 5 mg/kg BW LYC. The mice were firstly treated with LYC (Weikeqi-Biotech, Chengdu, China) by gavage, and then administered T-2 toxin by gavage 2 h later for 28 days. T-2 toxin was dissolved in a 4% ethanol solution, and LYC was dissolved in corn oil. Since the ethanol content in the final working solution was extremely low, deionized water was used in the solvent control group of the T-2 poisoning model. In the in vivo LYC experiment, corn oil was used as a delivery carrier to promote LYC absorption, so corn oil became the control treatment in the body. Existing studies and our preliminary experiments have confirmed that these solvents did not cause any adverse reactions in mice. Based on the size of testes in mice, 20 mice/group were used as the appropriate sample size [37,38]. It can provide adequate statistical power for the main observation indicators of this study while adhering to the 3R principles of laboratory animal ethics. If the premature death of animals results in the inability to collect behavioral and histological data, they were be excluded from the study. The body weight of each mouse was recorded weekly throughout the experiment. In this experiment, the dose of T-2 toxin and LYC selected for the mice was mainly based on the median lethal dose (LD50) and our preliminary studies [15,39,40]. After the trial, the testes were collected, photographed, and weighed. Serum and testicular tissues were used to assess circulating testosterone concentrations and related protein expression.

### 2.2. Testicular Histopathology Analysis

Testicular tissues collected from the animals were fixed using paraformaldehyde. Conventional paraffin embedding techniques were employed to prepare fixed testicular tissues. Tissue sections (5 μm) from each testicular segment underwent H&E staining (Servicebio, Wuhan, China) and were examined under an optical microscope [41]. Five sections per testis (*n* = 3) were examined for seminiferous tubules.

### 2.3. TM3 Cell Culture and Treatment

A total of 100 μg/mL streptomycin, 100 IU/mL penicillin (Sevenbio, Beijing, China), and 10% FBS were added to the culture medium (DMEM/F12, Servicebio, Wuhan, China) and cultured with the TM3 cell line (CRL-1714, ATCC, Manassas, VA, USA). TM3 cell culture took place within a humidified chamber maintained at 37 °C with 5% CO_2_ under constant-temperature conditions.

(1) For the LYC dose-screening cell model, LYC was initially dissolved in DMSO to obtain a 1 mM stock solution, which was then diluted in culture medium to the desired final concentrations. Different concentrations of LYC were applied to TM3 cells (0, 0.1, 0.5, 1, 5 or 10 μM) for 24 h. After that, cell viability was measured.

(2) For the T-2 toxin poisoning cellular model, TM3 cells were assigned to four groups: 0 nM: 0 nM T-2 toxin, 1 nM: 1 nM T-2 toxin, 5 nM: 5 nM T-2 toxin, 10 nM: 10 nM T-2 toxin. Following 24 h of treatment, the cells and culture supernatant were collected for further analyses.

(3) For the LYC-antagonized T-2 toxin cell model, four treatment groups were established using TM3 cells: CC (control group), TC (5 nM T-2 toxin group), TLC (5 nM T-2 toxin + 10 μM LYC group) and LC (10 μM LYC group). Following 24 h of treatment, the cells and culture supernatant were collected for further analyses.

(4) For the p38 molecular inhibition on the T-2 toxin cell model, four treatment groups were established using TM3 cells: control (control group), T-2 toxin (5 nM T-2 toxin group), SB203580 + T-2 (5 nM T-2 toxin + 3 μM SB203580 group) and SB203580 (3 μM SB203580 group). Following 24 h of treatment, the cells and culture supernatant were collected for further analyses.

(5) For the ROS scavenging with the T-2 toxin cell model, four treatment groups were established using TM3 cells: Con (control group), T-2 (5 nM T-2 toxin group), NAC + T-2 (5 nM T-2 toxin + 10 mM NAC) and NAC (10 mM NAC group). Following 24 h of exposure, TM3 cells and culture supernatant underwent collection for further analyses.

All the aforementioned pharmacological agents were dissolved in DMSO to prepare stock solutions. The final volumetric proportion of DMSO in all working treatment media was maintained below 0.1% (*v*/*v*).

### 2.4. TM3 Cell Viability Assay

TM3 cells were seeded into 96-well plates at a density of 1.5 × 10^4^ per well. Different doses of LYC, NAC and T-2 toxin were applied to TM3 cells according to the above procedure (*n* = 6). Then, freshly prepared CCK-8 reagent (100 μL, 10% final concentration in cell culture medium) was introduced into each well [39]. Following incubation at 37 °C without light for 1.5 h, the OD value was detected at 450 nm.

### 2.5. Immunofluorescence

TM3 cells were cultured in 24-well plates at a seeding density of 5 × 10^4^ cells per well. After treatment and PBS rinse, samples (*n* = 3) were fixed using 4% paraformaldehyde over 15 min. We subsequently permeabilized the samples over 10 min under room temperature using the enhanced immunostaining permeabilization reagent (Beyotime, Shanghai, China). After PBS rinsing, the samples underwent incubation using QuickBlock™ blocking buffer for immunological labeling (Beyotime, Shanghai, China) over 30 min at room temperature. 17β-HSD (ABclonal, Wuhan, China) primary antibodies (1:100) were subsequently added. Following incubation at room temperature, we removed the primary antibody, washed the samples, and then incubated them with a FITC-conjugated goat anti-rabbit secondary antibody (APExBIO, Houston, TX, USA). Incubation of the mixture took place for 1 h at room temperature without light. Following discard of the secondary antibody, DAPI solution was introduced and the samples were protected from light during incubation. Following PBS washing, target protein expression levels were visualized under a fluorescence microscope. Five random non-overlapping cortical fields per section were captured by two blind investigators.

### 2.6. Testosterone Content Determination

Testosterone levels in the TM3 cell culture medium (*n* = 3) underwent measurement as follows: following treatment of TM3 cells, collection of the culture medium took place, and after centrifugation at 1500 rpm for 5 min, the culture supernatant was collected for evaluation via an ELISA detection kit to measure the testosterone level [15].

For measurement of the serum testosterone level, blood was obtained from the mouse eyeballs and left to stand for 2 h at room temperature. Subsequently, the serum was centrifuged at 2500 rpm for 10 min under 4 °C, after which, the resulting supernatant was analyzed for testosterone content (*n* = 3) [42]. This ELISA assay (Elabscience, Wuhan, China) was conducted following the manufacturer’s protocol [15].

### 2.7. Molecular Docking

The LYC molecular structure was constructed via ChemDraw 22.0.0 (Revvity Signals Software, Waltham, MA, USA). The p38 3D structure was selected and reviewed via the UniProt database and downloaded from the AlphaFold protein structure database. By using AutoDoRck Vina 1.1.2 (The Scripps Research Institute, La Jolla, CA, USA), molecular binding docking between p38 and LYC was performed. The results were visualized via PyMOL 3.1 (DeLano Scientific LLC, San Carlos, CA, USA) and Ligplot 2.2 (European Molecular Biology Laboratory, Heidelberg, Germany).

### 2.8. Quantification of Oxidative Stress Indices

Commercial assay kits (Nanjing Jiancheng Bioengineering Institute, Nanjing, China) were employed to measure oxidative stress indicators: total antioxidant capacity (T-AOC; A015-1-1), superoxide dismutase (SOD; A001-2-1), malondialdehyde (MDA; A003-1-1), and catalase (CAT; A007-2-1), following the manufacturer’s protocol. Three independent biological experiments were performed.

### 2.9. Measurement of Intracellular Reactive Oxygen Species (ROS)

TM3 cells were seeded in 24-well plates at a density of 5 × 10^4^ cells/well. Following 24 h of exposure, the medium was removed, and cells underwent three PBS rinses. Following the manufacturer’s instructions, 200 μL ROS detection working solution was prepared using the ROS Assay Kit (S0033S, Beyotime Biotechnology, Shanghai, China) and added into each well. After incubation, the cells received three washes using PBS. Intracellular ROS levels underwent assessment by fluorescence intensity under an inverted fluorescence microscope. Five random non-overlapping cortical fields per section (*n* = 3) were captured by two blind investigators.

### 2.10. Quantitative RT-PCR

mRNA levels of testosterone synthase and key components of the MAPK pathway were quantified using quantitative reverse transcription polymerase chain reaction (qRT-PCR). After isolating mRNA out of TM3 cells along with mouse testicular tissue, a reverse transcription kit (+gDNA wiper) was used to synthesize complementary DNA (cDNA) (Vazyme, Nanjing, China). Gene expression was detected via qRT-PCR employing 2× Universal Blue SYBR Green qPCR Master Mix (Servicebio, Wuhan, China) using a qTOWER^®^ (Analytikjena, Jena, Germany) system. As a reference gene, β-actin was used as a control for quantifying the relative expression of genes through the 2^−△△CT^ methodology. Three independent biological experiments were performed. Table S1 shows primer sequences employed in this work.

### 2.11. Western Blot Analysis

Following lysis buffer treatment of the testicular tissue and TM3 cells, the mixtures were centrifuged at 12,000 rpm for 10 min at 4 °C, and the supernatant was harvested (*n* = 3). Determination of protein concentrations was performed with bovine serum albumin (BSA) serving as the standard, employing Coomassie Brilliant Blue reagent. Western blotting served to assess associated protein levels [43]. Table S2 displays the antibodies used in this test.

### 2.12. Data Analysis

Results are expressed as mean ± SD. Assumptions of normality and homogeneity of variance were evaluated prior to statistical analysis. Statistical comparisons were analyzed using one-way ANOVA, followed by either Tukey’s HSD test (for all pairwise intergroup comparisons) or Dunnett’s test (for comparisons of each treatment group versus the control group) for post hoc multiple comparisons. Data analysis and visualization were performed with GraphPad Prism 8.0 (GraphPad Software Inc., San Diego, CA, USA). Differences were considered statistically significant at *p* < 0.05. Statistical significance between groups was indicated by distinct symbols: asterisks (*) were used for comparisons against the normal control group, and hashes (#) were used for comparisons against the T-2 model group. The significance thresholds were set as * *p* < 0.05, ** *p* < 0.01 for control comparisons, and # *p* < 0.05, ## *p* < 0.01 for T-2 model group comparisons. The Metware Cloud platform (https://cloud.metware.cn), a free online tool for data analysis, was utilized to conduct Clustered Heatmap and Advanced Single-Omics Correlation Analysis.

## 3. Results

### 3.1. LYC Alleviates Testicular Injury and Testosterone Synthesis Disorders Caused by T-2 Toxin

**Figure 1:**
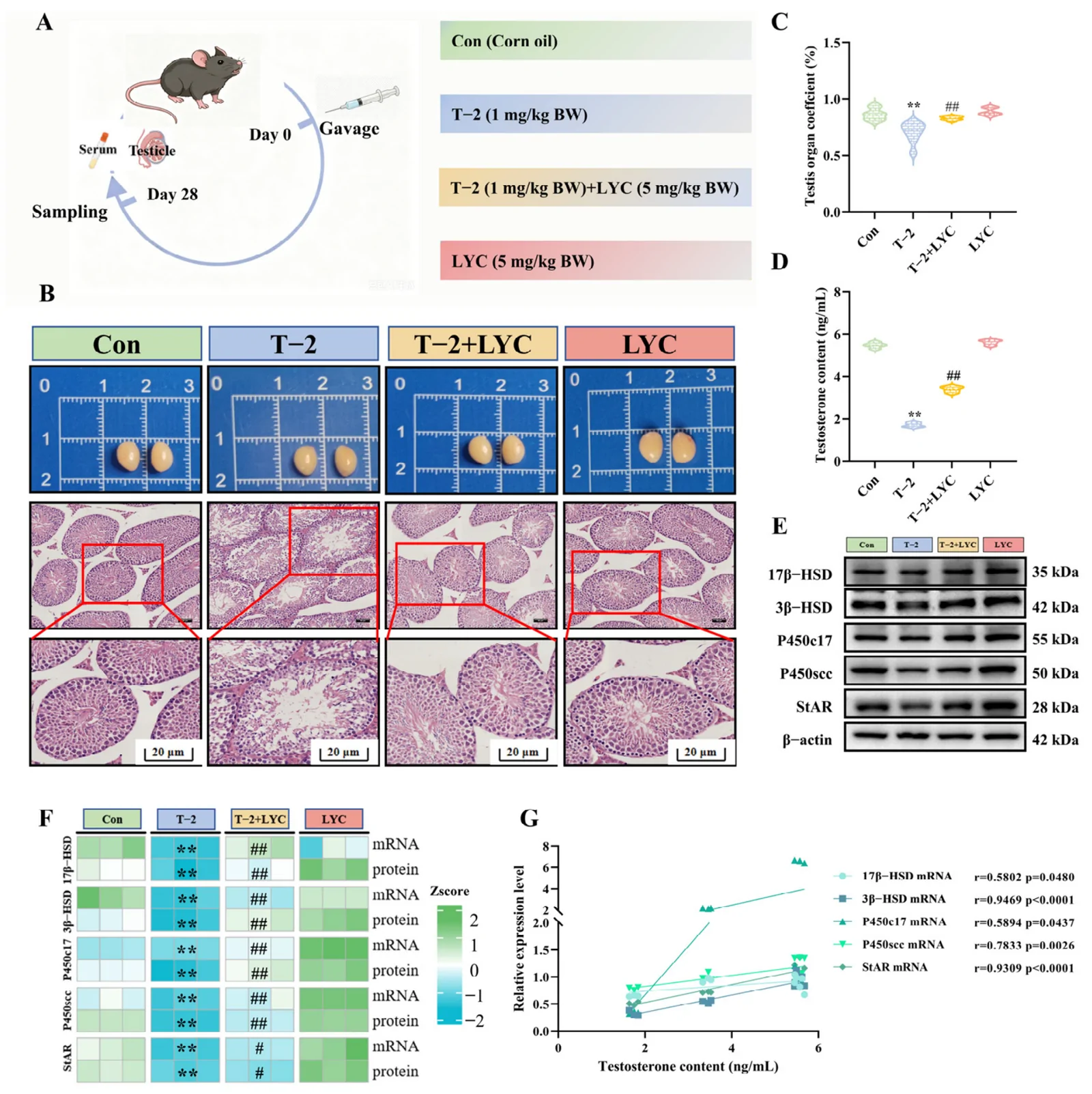
LYC inhibits testicular injury and testosterone synthesis disorder caused by T-2 toxin. (**A**) Establishment of the animal model; (**B**) testicular picture and H&E staining of testicular histology structure, 50 μm for low-power panels; 20 μm for high-power magnified panels; (**C**) testis organ coefficient; (**D**) serum testosterone content; (**E**) Western blotting bands of 17β-HSD, 3β-HSD, P450c17, P450scc and StAR; (**F**) the relative mRNA and protein expression levels of the key steroidogenic enzymes 17β-HSD, 3β-HSD, P450c17, P450scc, and StAR; (**G**) correlation analysis of serum testosterone level and testosterone synthase expression levels. Experimental data are reported as the means ± SDs. Asterisk for the significant differences between the Con group and another group, ** *p* < 0.01. Hashes for significant difference between T-2 group and T-2 + LYC group, # *p* < 0.05, ## *p* < 0.01.

**Figure 2:**
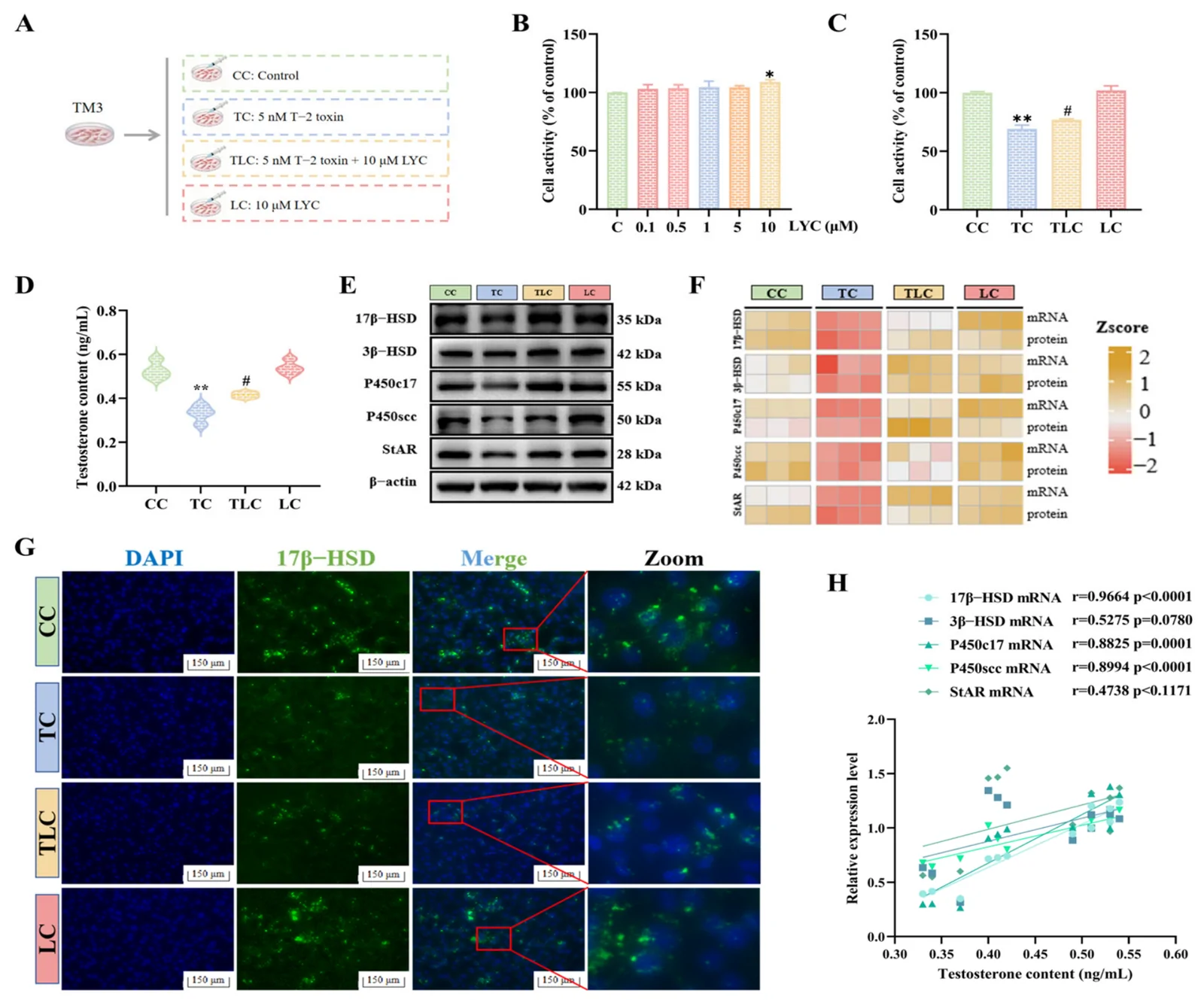
LYC alleviates the inhibition of testosterone secretion caused by T-2 toxin in TM3 cells. (**A**) TM3 cell treatment model; (**B**) TM3 cells viability after treated with 0.1, 0.5, 1, 5, and 10 μM LYC for 24 h; (**C**) TM3 cells viability under 5 nM T-2 toxin exposure with/without 10 μM LYC for 24 h; (**D**) testosterone levels in the culture medium of TM3 cells; (**E**) Western blotting bands of 17β-HSD, 3β-HSD, P450c17, P450scc, and StAR; (**F**) the relative mRNA and protein expression levels of 17β-HSD, 3β-HSD, P450c17, P450scc, and StAR in TM3 cells; (**G**) 17β-HSD immunofluorescence in TM3 cells; (**H**) correlation analysis of testosterone content in the supernatant of TM3 cells and expression of testosterone synthases. Experimental data are reported as the means ± SDs. Asterisk for the significant differences between the CC group and another group, * *p* < 0.05, ** *p* < 0.01. Hashes for the significant difference between TC group and TLC group, # *p* < 0.05.

This study sought to determine whether LYC protects against T-2 toxin-triggered testicular tissue damage and testosterone synthesis disorders in mice (see Figure 1A). Mice received both T-2 toxin and LYC. T-2 toxin exposure caused atrophied seminiferous tubules, disorganized spermatogenic cells in the seminiferous epithelium, and very few spermatozoa in the lumen, whereas this injury showed a significant reduction in the T-2 + LYC group (see Figure 1B). Relative to the Con group, mice receiving T-2 toxin exhibited a notable reduction in testis organ coefficient and serum testosterone levels, which was restored in the T-2 + LYC group (*p* < 0.01) (see Figure 1C,D). As shown in Figure 1E,F, T-2 toxin exposure significantly reduced the gene and protein levels of the testosterone synthases (*p* < 0.01). However, LYC treatment significantly increased the gene and protein levels of the testosterone synthases compared with the T-2 group (*p* < 0.05). At the same time, the correlation analysis between the serum testosterone level and testosterone synthase expression levels showed that there was a significant positive correlation between testosterone content and testosterone synthase expression (see Figure 1G). The results indicated that LYC could alleviate T-2 toxin-induced testicular injury and testosterone synthesis disorders in mice.

### 3.2. LYC Alleviates the Testosterone Synthesis Disorders Caused by T-2 Toxin in TM3 Cells

Firstly, we examined the effect of LYC on the activity of TM3 cells. It was found that 10 μM LYC markedly enhanced the cell viability (*p* < 0.05) (see Figure 2A,B). And cell viability was notably greater in the TLC group compared with that in the TC group (*p* < 0.05) (see Figure 2C). Therefore, 10 μM LYC was selected as the treatment concentration for subsequent investigations. Figure 2D shows a significant decrease in testosterone concentrations in the culture medium after exposure to T-2 toxin (*p* < 0.01). Conversely, co-administration of LYC and T-2 toxin markedly increased testosterone concentrations (*p* < 0.05). Furthermore, the TC group significantly reduced the gene and protein levels of the testosterone synthase in TM3 cells (*p* < 0.01). However, LYC treatment significantly increased the gene and protein levels of the testosterone synthases compared with the TC group (see Figure 2E,F). Immunofluorescence analysis showed a decrease in green fluorescence intensity for 17β-HSD following T-2 toxin exposure compared with the CC group. However, the TLC group markedly enhanced the green fluorescence intensity of 17β-HSD relative to the TC group (see Figure 2G). The correlation analysis of testosterone content in the TM3 cell supernatant and the expression of testosterone synthases simultaneously confirmed that LYC affected the expression of testosterone synthases and altered the testosterone content (see Figure 2H). These findings indicated that LYC alleviated T-2 toxin-caused testosterone synthesis impairment in TM3 cells.

### 3.3. LYC Inhibits T-2 Toxin-Induced MAPK Pathway Activation in Testis and TM3 Cells

**Figure 3:**
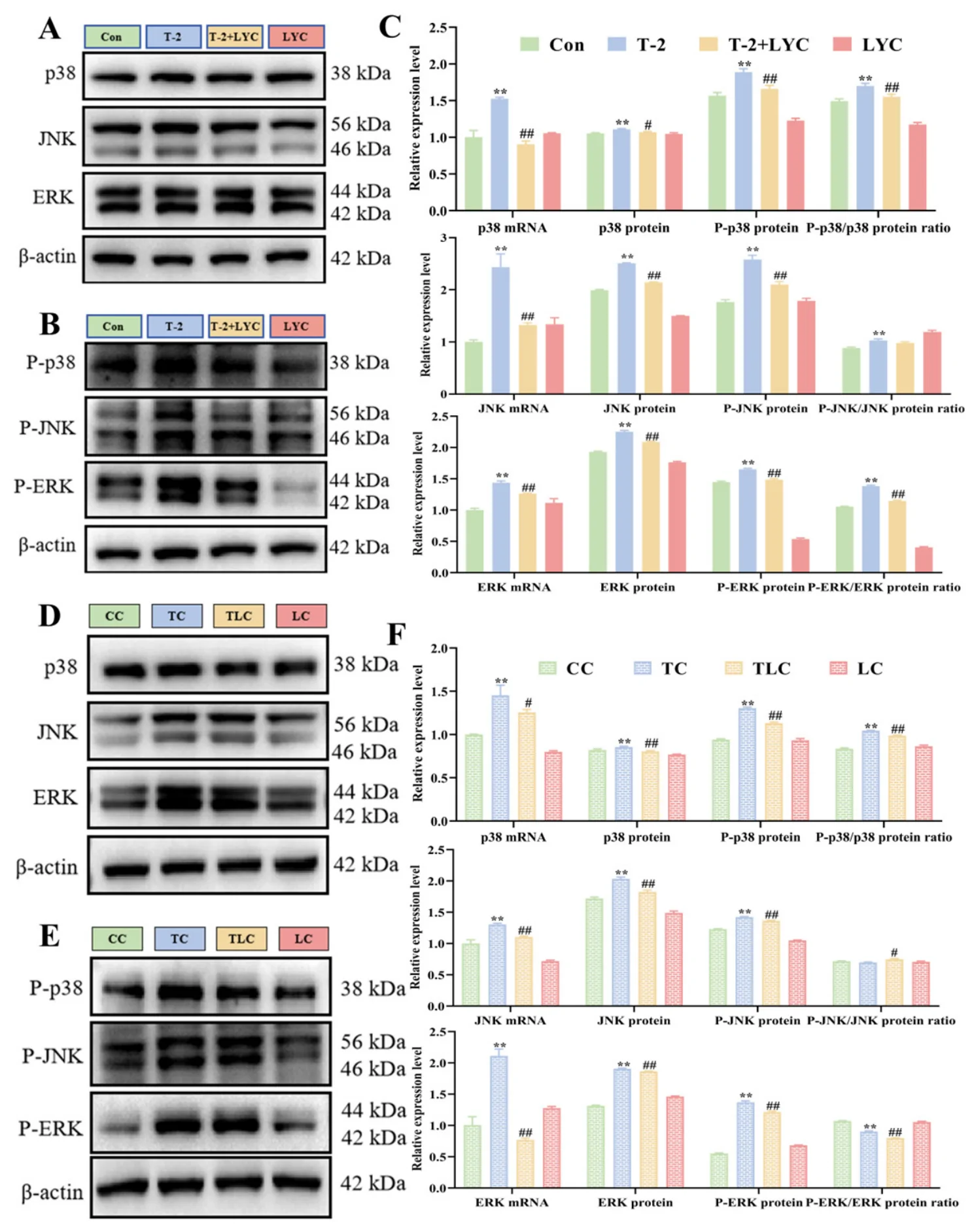
LYC inhibits T-2 toxin-induced MAPK pathway activation in mouse testes and TM3 cells. (**A**) Western blotting bands of p38, JNK and ERK in mouse testes; (**B**) Western blotting bands of P-p38, P-JNK and P-ERK in mouse testes; (**C**) the relative mRNA and protein expression levels of p38, JNK, and ERK in mouse testes; (**D**) Western blotting bands of p38, JNK and ERK in TM3 cells; (**E**) Western blotting bands of P-p38, P-JNK and P-ERK in TM3 cells; (**F**) the relative mRNA and protein expression levels of p38, JNK, and ERK in TM3 cells. Experimental data are reported as the means ± SDs. Asterisk for the significant differences between the control and another group, ** *p* < 0.01. Hashes for the significant difference between T-2 group and T-2 + LYC group or TC group and TLC group # *p* < 0.05, ## *p* < 0.01.

Relative to the Con group, T-2 toxin exposure significantly upregulated the mRNA, protein (p38, JNK, and ERK) and phosphorylation levels (P-p38, P-JNK, P-ERK) of MAPK pathway-associated factors (*p* < 0.01) (see Figure 3A–C). The elevated phosphorylation levels of these core kinases are the canonical hallmark of MAPK signaling pathway activation. Conversely, LYC administration markedly decreased MAPK pathway-associated factors in the mRNA, protein and phosphorylation levels versus the T-2 group (*p* < 0.05) (see Figure 3A–C). TM3 cells further corroborated the consistent inhibitory effect of LYC on T-2 toxin-caused MAPK pathway stimulation (*p* < 0.05) (see Figure 3D–F). The results demonstrated that LYC effectively prevented T-2 toxin-induced activation of the MAPK pathway both in testis and TM3 cells.

### 3.4. T-2 Toxin Inhibits the TORC2/CREBAxis via Activating p38 Molecule

**Figure 4:**
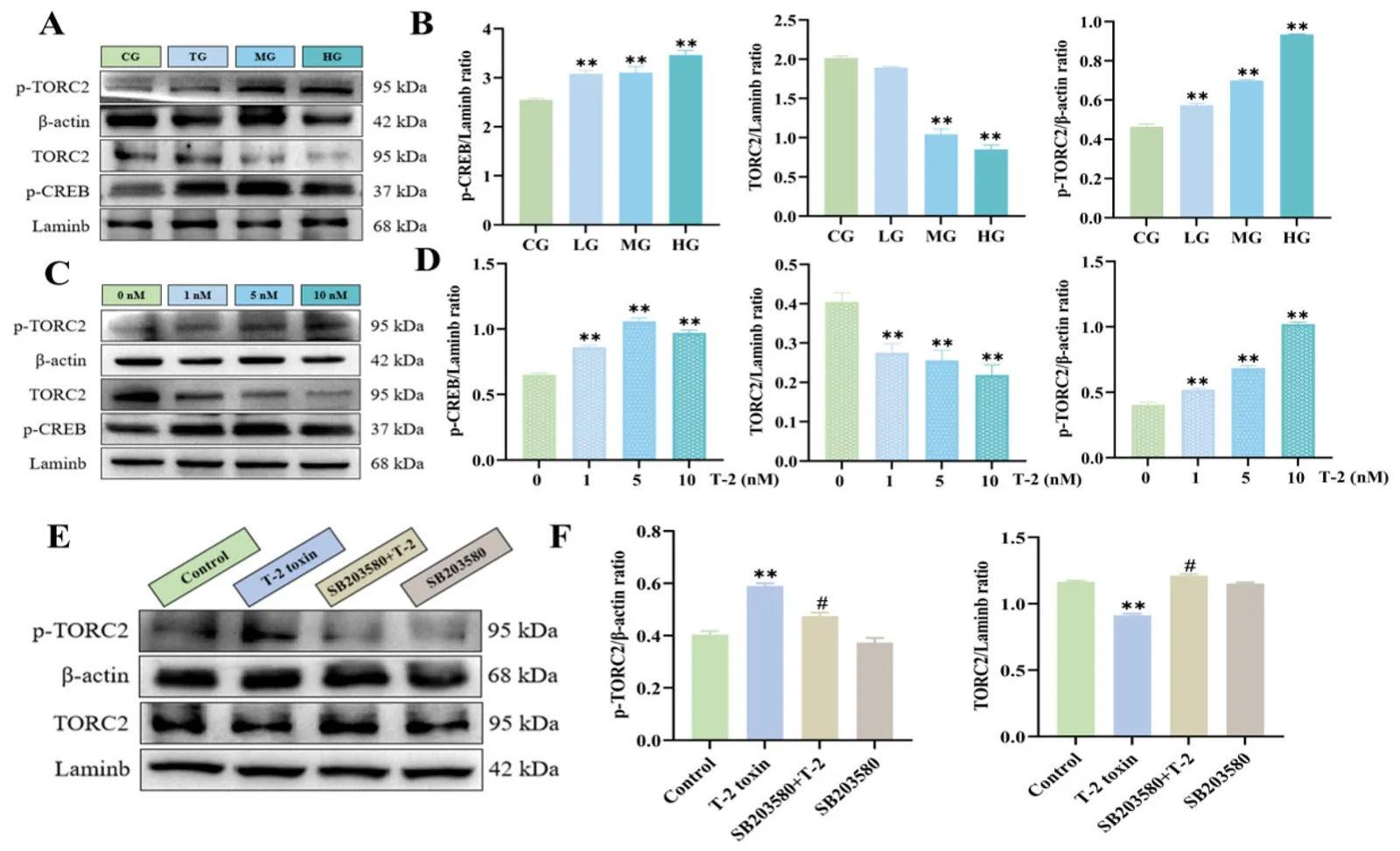
T-2 toxin activates the p38/TORC2/CREB signaling axis in mouse testes and TM3 cells. (**A**) Western blotting bands of cytoplasmic p-TORC2, nuclear TORC2, and nuclear p-CREB in mouse testes of the T-2 toxin poisoning model; (**B**) the protein expression levels of cytoplasmic p-TORC2, nuclear TORC2, and nuclear p-CREB in mouse testes of the T-2 toxin poisoning model; (**C**) Western blotting bands of cytoplasmic p-TORC2, nuclear TORC2, and nuclear p-CREB in TM3 cells of the T-2 toxin poisoning model; (**D**) the protein expression levels of cytoplasmic p-TORC2, nuclear TORC2, and nuclear p-CREB in TM3 cells of the T-2 toxin poisoning model; (**E**) Western blotting bands of cytoplasmic p-TORC2, nuclear TORC2, and nuclear p-CREB in mouse testes of the SB203580 + T-2 toxin intervention model; (**F**) the protein expression levels of cytoplasmic p-TORC2, nuclear TORC2 in mouse testes of the SB203580 + T-2 toxin intervention model. Experimental data are reported as the means ± SDs. Asterisk for the significant differences between the control and another group, ** *p* < 0.01. Hashes for the significant difference between T-2 group and SB203580 + T-2 group # *p* < 0.05.

As shown in Figure 4A–D, T-2 toxin exposure markedly increased nuclear p-CREB and cytoplasmic p-TORC2 protein levels while decreasing nuclear TORC2 in both testicular tissue and TM3 cells (*p* < 0.01), indicating that T-2 toxin inhibited the TORC2/CREB signaling axis; co-treatment with 3 μM SB203580 and 5 nM T-2 toxin upregulated nuclear TORC2 and downregulated cytoplasmic p-TORC2 versus T-2 toxin alone (*p* < 0.05) (see Figure 4E,F), demonstrating that T-2 toxin disrupted testosterone production through the p38/TORC2/CREB signaling axis.

### 3.5. ROS Mediates T-2 Toxin-Induced Activation of the p38/TORC2/CREB Axis

**Figure 5:**
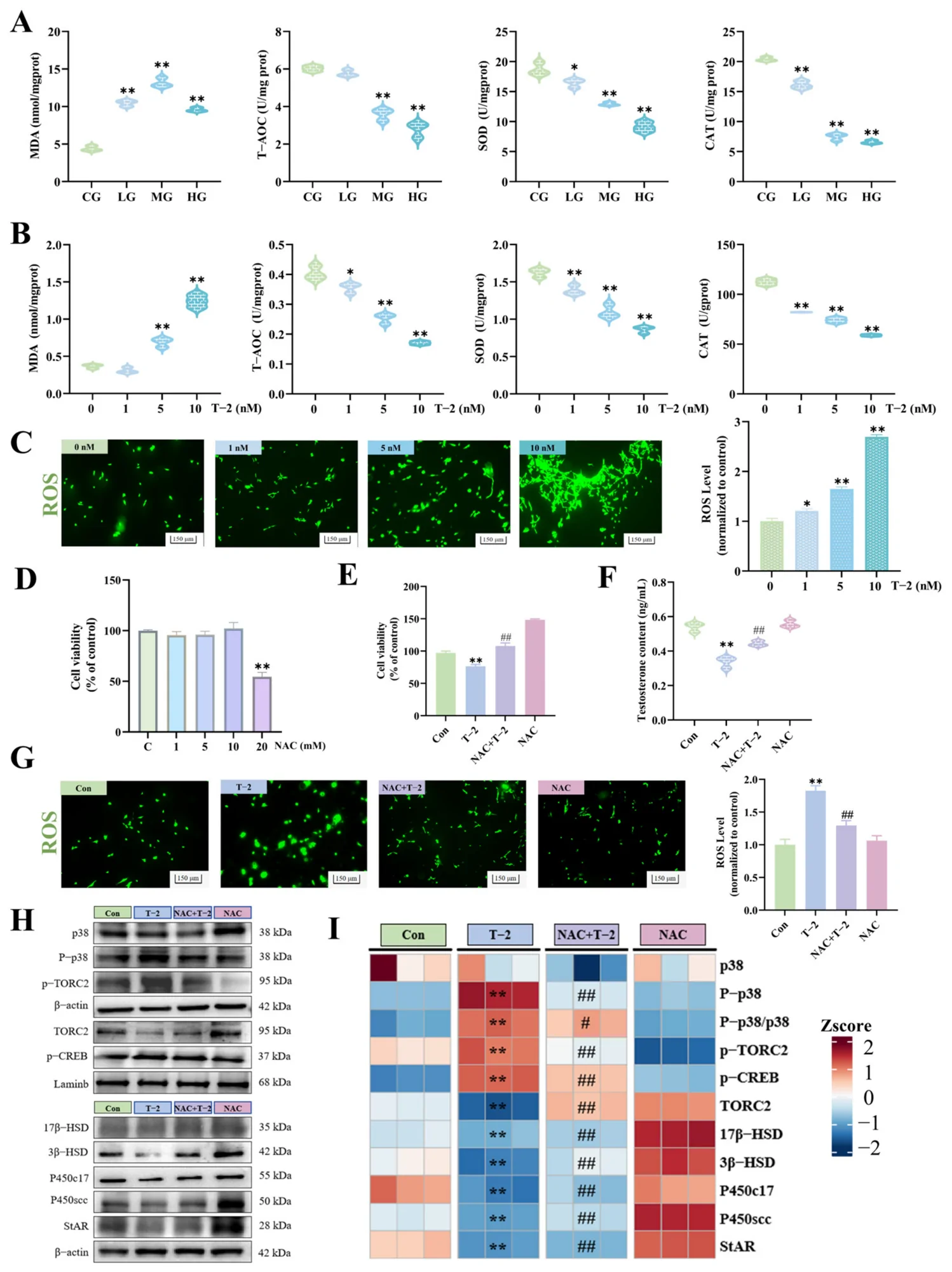
ROS mediates T-2 toxin-induced activation of the p38/TORC2/CREB axis. (**A**) MDA content, T-AOC content, SOD activity, and CAT activity in the mouse testis tissue of the T-2 toxin poisoning model; (**B**) MDA content, T-AOC content, SOD activity, and CAT activity in TM3 cells of the T-2 toxin poisoning model; (**C**) ROS fluorescence intensity in TM3 cells of the T-2 toxin poisoning model; (**D**) activity of TM3 cells treated with 1, 5, 10 and 20 mM NAC for 24 h; (**E**) activity of TM3 cells after 24 h of 5 nM T-2 toxin antagonism by 10 mM NAC; (**F**) testosterone levels in the culture supernatant of TM3 cells from the NAC + T-2 toxin intervention model; (**G**) ROS fluorescence intensity in TM3 cells of the NAC + T-2 toxin intervention group; (**H**) representative Western blot bands of p38/TORC2/CREB/StAR axis-related molecules in TM3 cells from the NAC + T-2 toxin intervention model; (**I**) relative expression levels of p38/TORC2/CREB/StAR axis-related molecules in TM3 cells from the NAC + T-2 toxin intervention model. Experimental data are reported as the means ± SDs. Asterisk for the significant differences between the control and another group, * *p* < 0.05, ** *p* < 0.01. Hashes for the significant difference between T-2 group and NAC + T-2 group # *p* < 0.05, ## *p* < 0.01.

T-2 toxin administration dose-dependently increased MDA and ROS levels but reduced T-AOC, SOD, and CAT activities both in testicular tissue and TM3 cells (*p* < 0.05) (see Figure 5A–C). To determine whether ROS acts as a direct upstream signal in T-2 toxin-induced p38/TORC2/CREB axis activation, ROS scavengers (NAC) were employed in this study. The optimal NAC concentration for TM3 cells was first established, with 10 mM NAC showing little effect on cell viability (*p* > 0.05) (see Figure 5D). The T-2 + NAC group markedly improved TM3 cell viability relative to the T-2 group (*p* < 0.01) (see Figure 5E). The data in Figure 5F indicate that a significant decrease in testosterone levels in the cell culture medium occurred following treatment with T-2 toxin (*p* < 0.01). In contrast, co-treatment with NAC and T-2 toxin led to a marked elevation in testosterone concentrations (*p* < 0.01). In the T-2 + NAC group, ROS levels were significantly reduced, p38 and P-p38 expression decreased, nuclear TORC2 expression increased, cytoplasmic p-TORC2 and nuclear p-CREB expression decreased, and key testosterone biosynthetic enzymes (17β-HSD, 3β-HSD, P450scc, P450c17, and StAR) showed increased protein expression versus T-2 toxin alone (*p* < 0.05) (see Figure 5G–I). Taken together, our results demonstrate T-2 toxin-triggered testosterone biosynthesis impairment occurs via the ROS/p38/TORC2/CREB axis.

### 3.6. LYC Inhibiting ROS/p38/TORC2/CREB Axis Induced by T-2 Toxin in Testis and TM3 Cells

**Figure 6:**
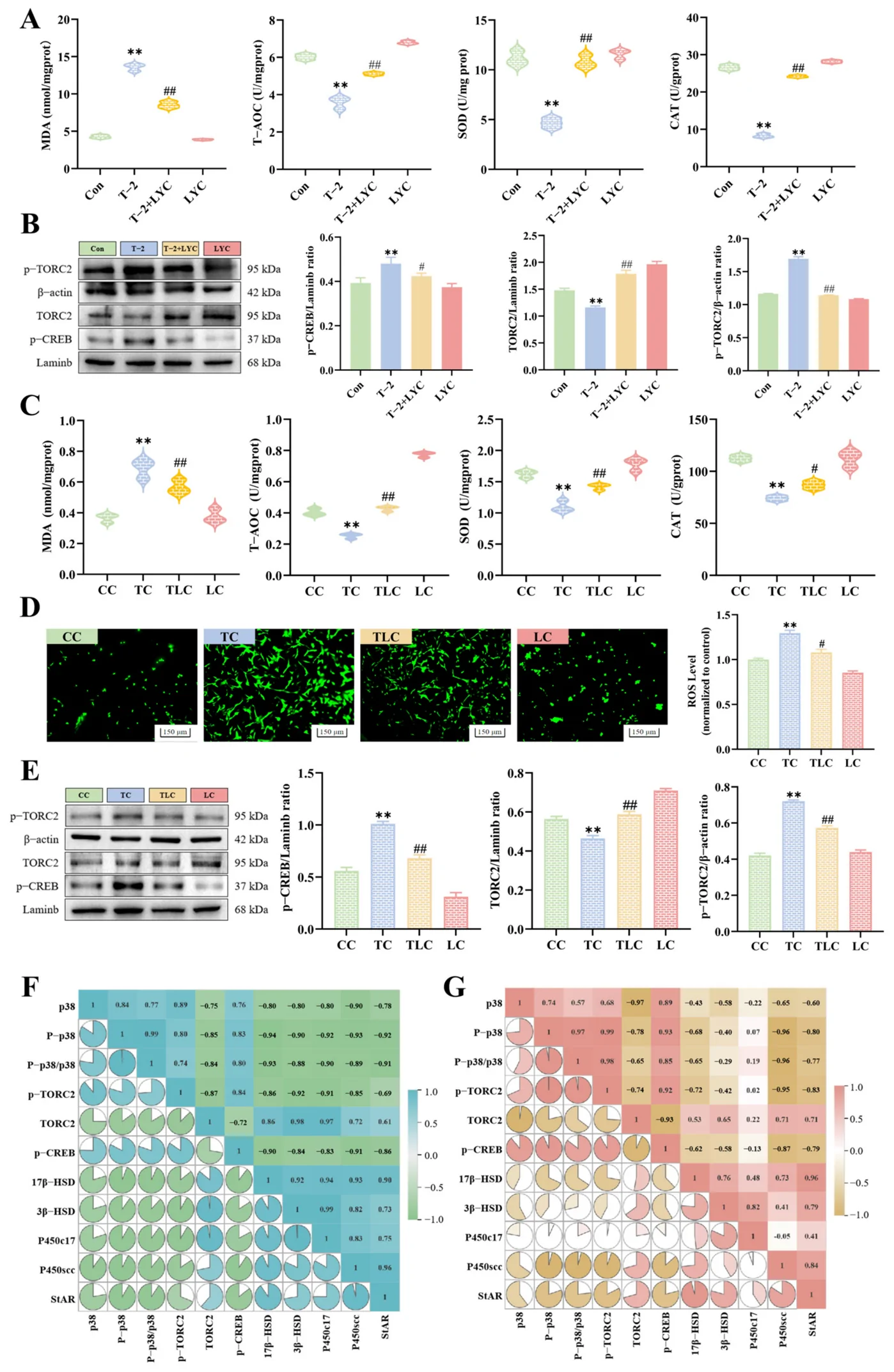
LYC inhibits T-2 toxin exposure-induced ROS/p38/TORC2 axis activation. (**A**) MDA content, T-AOC content, SOD activity, and CAT activity in the mouse testis tissue of the LYC + T-2 toxin intervention model; (**B**) Western blotting expression level of p-CREB, p-TORC2 and TORC2 in mouse mode of LYC intervention; (**C**) MDA content, T-AOC content, SOD activity, and CAT activity in the TM3 cells of the LYC + T-2 toxin intervention model; (**D**) ROS fluorescence intensity in TM3 cells of the LYC + T-2 toxin intervention model; (**E**) Western blotting expression level of p-CREB, p-TORC2 and TORC2 in cell model of LYC intervention; (**F**) correlation analysis of molecules in the p38/TORC2/CREB/StAR signaling axis in testicular tissue of mice from the LYC + T-2 toxin intervention model; (**G**) correlation analysis of molecules in the p38/TORC2/CREB/StAR signaling axis in testicular tissue of TM3 cells from the LYC + T-2 toxin intervention model. Experimental data are reported as the means ± SDs. Asterisk for the significant differences between the control and another group, ** *p* < 0.01. Hashes for the significant difference between T-2 group and T-2 + LYC group or TC group and TLC group # *p* < 0.05, ## *p* < 0.01.

As illustrated in Figure 6, the T-2 + LYC or TLC group resulted in a notable reduction in MDA, whereas T-AOC, SOD, and CAT were significantly elevated compared with the T-2 or TC group (*p* < 0.05) (see Figure 6A,C). The TLC group notably reduced intracellular ROS levels relative to the TC group (*p* < 0.05) (Figure 6D). Both the T-2 + LYC and TLC group exhibited significantly decreased p38 and P-p38 protein levels concurrently compared with the T-2 or TC group (*p* < 0.05) (see Figure 3C,E). In addition, cytoplasmic p-TORC2 and nuclear p-CREB protein expression was downregulated, while nuclear TORC2 expression was upregulated between T-2 and T-2 + LYC or TC and TLC (*p* < 0.05) (see Figure 6B,E). A significant inverse relationship was found between the p38/TORC2/CREB pathway and testosterone synthesis in LYC-treated T-2 toxin exposure models (see Figure 6G). Simulating the molecular docking between LYC and p38 revealed that LYC can target p38 (Figure S1A). Additionally, immunofluorescence staining revealed a marked elevation of P-p38 green fluorescence in the CC group compared with TC group. Compared with the TC group, the intensity of the green fluorescence of P-p38 in the TLC group was reduced (Figure S1B). Taken together, LYC counteracts T-2 toxin-induced ROS/p38/TORC2/CREB axis activation, thereby mitigating testosterone synthesis impairment.

## 4. Discussion

Contamination of feed and food by T-2 toxin poses serious health risks for animals as well as humans worldwide [44,45]. T-2 toxin causes toxic effects across various organ systems, particularly the reproductive system. Research has shown that the subacute poisoning of rabbits by T-2 toxin results in sperm malformations, Leydig cell impairment, abnormal sperm motility increase, and testosterone level decline [46]. T-2 toxin inhibited hCG and caused a decrease in testosterone production via downregulating steroidogenesis enzyme mRNA expression, as well as the activities of steroidogenesis enzymes [47]. Earlier work from our group demonstrated T-2 toxin exposure reduced reproductive organ size, body weight, and daily sperm production while increasing the sperm malformation rate in mice [13]. Furthermore, maternal T-2 toxin exposure not only compromised the testicular antioxidant system in male offspring but also impaired their testosterone synthesis during juvenile development [48]. These findings indicate that T-2 toxin may have a significant impact on male fertility by reducing the function of testosterone synthesis in the testes and increasing abnormal sperm production. LYC is a safe and effective carotenoid that is found mainly in tomatoes [49]. LYC has antioxidant, hypolipidemic, anticancer, immunomodulatory, hepatoprotective, hypoglycemic, cardiovascular protective, anti-inflammatory, reproductive protective, neuroprotective and osteoporosis inhibitory effects [47]. Owing to its high nutritional value, LYC is recognized as a food additive in more than 50 countries and is widely utilized in health foods, pharmaceuticals, cosmetics, agricultural products, and other areas [50]. Some studies have indicated that LYC can improve male reproductive performance by increasing sperm motility, the sperm count, and testosterone levels [51]. Accumulating studies have confirmed that T-2 toxin exposure severely disrupts testosterone synthesis and causes male reproductive damage, whereas whether LYC could alleviate T-2 toxin-induced male reproductive injury remains unclear. Therefore, this study was designed to explore the protective effect and underlying mechanism of LYC against T-2 toxin-triggered testosterone synthesis disorder and male reproductive impairment. The present results found that T-2 toxin treatment significantly impaired testosterone synthesis in both TM3 Leydig cells and mice. Further mechanistic exploration demonstrated that the activation of the ROS/p38/TORC2/CREB signaling axis was involved in T-2 toxin-induced testosterone biosynthesis disorder. LYC supplementation could effectively reverse the above adverse toxic effects, thereby exerting a protective role against T-2 toxin-caused male reproductive damage.

Testosterone is the predominant androgen and is essential for regulating male physiology. Testosterone plays a significant role in promoting spermatogenesis, developing male reproductive organs, maintaining secondary sexual characteristics, and influencing libido and sexual behavior [52,53,54]. Numerous studies have indicated that LYC supplementation is beneficial for male reproductive health. Research has shown that LYC is involved in and can be depleted during the process of testosterone synthesis in the testis [55]. Moreover, LYC supplementation alleviated male reproductive disorders caused by various exogenous substances. LYC has been reported to attenuate DEHP-induced decline in testosterone synthesis in Leydig cells and secretion dysfunction in Sertoli cells [39]. Furthermore, LYC mitigated benzo[a]pyrene (BaP)-induced changes in sperm morphology, decreased testosterone levels and reduced germ cell apoptosis in mice [56]. In this study, T-2 toxin treatment resulted in marked reductions in testis coefficient and germ cell count, along with testosterone levels, as well as disorganization of testicular histoarchitecture. However, adding LYC can significantly reverse the damage to the reproductive system caused by T-2 toxin, the decline in the number and activity of reproductive cells, and even the decline in related functions such as testosterone synthesis and spermatogenesis. Testosterone is primarily synthesized by Leydig cells, and dysfunction of these cells critically contributes to impaired testosterone synthesis. T-2 toxin treatment markedly reduced both the viability and testosterone secretion of TM3 cells. However, the addition of LYC significantly reversed these changes. These results indicate that LYC can alleviate the decline in the survival rate of TM3 cells and the reduction in testosterone synthesis caused by T-2 toxin. To examine whether LYC ameliorates T-2 toxin-triggered testosterone synthesis disorder, the levels of testosterone synthesis-related proteins were assessed. StAR facilitates cholesterol translocation across the mitochondrial membranes in Leydig cells. CYP11A1 converts cholesterol to pregnenolone, which is further processed to testosterone in the endoplasmic reticulum via sequential reactions catalyzed by 3β-HSD, CYP17A1, and 17β-HSD [57]. Changes in testosterone synthase levels can significantly affect testosterone synthesis. Previous work demonstrated T-2 toxin suppressed testosterone production in Leydig cells through attenuating steroidogenic enzyme activity [26]. The aforementioned studies underscore the importance of maintaining testosterone-synthesizing enzyme levels, an essential element for mitigating T-2 toxin-triggered testosterone synthesis. LYC successfully reversed the DBP-induced declines in testosterone-synthesizing enzyme levels and testosterone content [32]. In agreement with the above studies, our data showed that LYC upregulated both protein and mRNA expression of testosterone synthase in T-2 toxin-treated mice and TM3 cells. Our findings suggest that LYC attenuates T-2 toxin-caused impairment of testosterone production and upregulates testosterone-synthesizing enzyme expression.

MAPK signaling is crucial for mediating T-2 toxin toxicity. Previous studies have found that the T-2 toxin leads to germ cell apoptosis by triggering an ROS-mediated MAPK cascade reaction [58,59]. Our previous research also revealed that the activation of the MAPK pathway mediates the barrier disruption of IPEC-J2 cells caused by T-2 toxin [44]. Some T-2 toxin antagonistic drugs also exert therapeutic effects through the MAPK signaling pathway. For instance, luteolin (BA) can alleviate the oxidative damage to the spleen caused by T-2 toxin through three mechanisms: reducing ROS, inhibiting MAPK signal transduction, and upregulating the Nrf2 pathway [60]. Collectively, the above findings demonstrate that the MAPK signaling pathway is involved in T-2 toxin-mediated toxic effects, and intervention targeting the MAPK signaling pathway may represent an effective strategy for counteracting T-2-toxin-induced toxicity. Luo et al. found that blocking p38 MAPK delays Leydig cell senescence, upregulates testosterone synthase, and promotes testosterone secretion in aged mice [61]. Selenium nanoparticles (Nano-Se) antagonize NiSO4-induced impairment of testosterone production in Leydig cells via inhibition of the miR-708-5p/p38 MAPK pathway [62]. Previous studies have shown that LYC is a potential inhibitor of p38. Earlier work has demonstrated that LYC suppresses p38, ERK, JNK, and NF-κB p65 signaling in eWAT derived from high-fat-diet-induced obese mice [63,64]. LYC exerts inhibitory effects on growth and promotes apoptosis in gastric cancer cells by suppressing EGFR/Ras/ERK along with p38 MAPK pathways, which are stimulated via ROS and NF-κB-driven COX-2 expression [65]. LYC attenuated TD-triggered MAPK pathway activation in rat livers [35]. Some research found that LYC can also protect the reproductive system by mitigating impaired testosterone synthesis ability induced by hazardous substances/pollutants [39,66]. However, most of these studies focused on regulating oxidative stress and cell death and did not focus on the regulatory role of MAPK signaling pathways [40]. Whether LYC attenuates T-2 toxin-triggered testosterone synthesis disruption through MAPK pathway inhibition remains to be elucidated. In our previous work, we evaluated how T-2 toxin affects MAPK signaling and found it raised protein levels of JNK, ERK, and P38. Application of SP600125, SCH772984, or SB203580 to suppress JNK, ERK, and p38 molecule activation revealed that inhibiting p38 alone can alleviate the reproductive toxicity of T-2 toxin. It is proved that p38 serves as a key target for T-2 toxin in male reproductive toxicity [15]. However, it remains unclear how activated p38 contributes to T-2 toxin-induced impairment of testosterone production. Previous studies have demonstrated that p38 phosphorylates TORC2, leading to its dissociation from p-CREB, thereby reducing StAR transcriptional activity and inhibiting testosterone synthesis [23]. We therefore postulated that the p38/TORC2/CREB axis mediated T-2 toxin-triggered testosterone biosynthesis impairment. In this study, we found that T-2 toxin phosphorylates TORC2 via p38, thereby reducing its nuclear localization. Combined with our previous research that inhibition of the p38 molecule mitigated the reduction in StAR transcription caused by T-2 toxin [15], we propose that targeting the p38/TORC2/CREB axis represents a promising strategy for alleviating T-2 toxin-triggered testosterone synthesis disruption. Nevertheless, the present study still has certain limitations. Although our results preliminarily clarified the sequential regulatory relationship among p38, TORC2, and CREB during T-2 toxin exposure, further specific and rigorous experiments, including site-directed mutagenesis, chromatin immunoprecipitation (ChIP), and luciferase reporter assays, are still required to fully validate the direct causal interaction between these molecules. These detailed mechanistic validations will be systematically explored in our future investigations.

Although T-2 toxin triggers testosterone synthesis disorders through activating the p38/TORC2/CREB axis, the answer to a subsequent key question, how T-2 toxin activates p38, is still unknown. Given that ROS accumulation is a characteristic of T-2 toxin toxicity and a key upstream signal for MAPK activation, we utilized the ROS scavenger NAC to detect whether p38 can still be activated under ROS depletion conditions and determine whether ROS acts as a pivotal mediator of T-2 toxin-triggered p38 activation. ROS depletion significantly attenuated T-2 toxin-triggered testosterone synthesis disorders and p38/TORC2/CREB axis activation. Collectively, our research results indicate that the activation of the p38/TORC2/CREB axis after T-2 toxin treatment is achieved through the accumulation of ROS. Here, we identify for the first time the intact ROS/p38/TORC2/CREB signaling axis as a key mediator of this pathological process, filling a critical mechanistic gap between upstream oxidative stress and downstream testosterone biosynthetic dysfunction. To identify safe and effective substances, LYC, which has a clear reproductive protection effect and ROS scavenging capacity, is a candidate drug. In this study, it is confirmed that LYC alleviated T-2 toxin-mediated disruption of testosterone biosynthesis by repressing the ROS/p38/TORC2/CREB axis. The present study demonstrates that LYC confers reproductive protection by precisely modulating this full signaling cascade, thereby anchoring its reproductive benefit to a clearly defined molecular mechanism. Furthermore, the signaling axis identified herein provides a valuable framework for mechanistic investigations and target screening of reproductive injury caused by other fusarium toxins, extending the translational relevance of our findings beyond a single mycotoxin. The inhibitory effect of LYC on p38 was mediated through two distinct mechanisms. On one hand, LYC exerted its potent antioxidant capacity, thereby attenuating the ROS burst triggered by T-2 toxin. Additionally, molecular docking simulations demonstrated that LYC could directly bind to p38. The molecular docking results revealed that LYC could target p38, and the immunofluorescence results revealed that LYC reduced the abundance of the P-p38 protein, suggested that LYC may inhibit the phosphorylation of p38 and hinder its activation by binding to p38. Additionally, LYC downregulated the mRNA and protein expression of JNK and ERK, along with p38, as well as phosphorylating JNK, ERK, and p38 (P-JNK, P-ERK, and P-p38), in TM3 cells and mouse testes treated with T-2 toxin. Given both JNK and ERK were also involved in the regulation of testosterone synthesis [18,67], this study revealed that LYC exerts regulatory effects on ERK and JNK pathways during the T-2 toxin challenge. Collectively, our inhibitor-based results support the contribution of the ROS/p38/TORC2/CREB axis to LYC-conferred protection against T-2-toxin-induced testosterone synthesis impairment. Nevertheless, these findings do not establish this as the sole underlying mechanism. LYC may also confer protective benefits through JNK- and ERK-related signaling. The exact functional roles of JNK and ERK, especially their modulation of testosterone-synthesizing enzyme expression, remain to be further elucidated.

This study has several limitations. The single LYC dosage used lacks dose–response validation and testicular accumulation data, and the translational relevance of its nutritional application remains unclear due to undefined bioavailability and physiologically attainable concentrations [68,69]. In addition, p38 MAPK verification depends only on pharmacological inhibition, and TM3 cell models cannot fully mimic the testicular microenvironment [70,71,72,73,74]. Future work will optimize LYC administration, conduct genetic validation, adopt more physiological models, and evaluate LYC bioavailability to strengthen its translational potential.

## 5. Conclusions

T-2 toxin exposure induced testicular testosterone biosynthesis disorders by activating the ROS/p38/TORC2/CREB signaling axis. LYC effectively repressing the ROS/p38/TORC2/CREB signaling axis, alleviating T-2 toxin-triggered testosterone biosynthesis disorders. This work offers a theoretical framework for mitigating T-2 toxin-induced reproductive toxicity along with novel insights into dietary interventions against mycotoxin-related male reproductive disorders.

## Data Availability

The original contributions presented in this study are included in the article/Supplementary Materials. Further inquiries can be directed to the corresponding author.

## Supplementary Materials

The following supporting information can be downloaded at: https://www.mdpi.com/article/10.3390/antiox15091060/s1, Table S1: Primer sequence and length of destination; Table S2: Antibodies required for Western Blot assay; Figure S1: LYC inhibited the activation of the p38-MAPK signaling pathway. (A) Simulated molecular docking between LYC and p38; (B) TM3 cells P-p38 immunofluorescence.
