# Supplementary material for: Lycopene Alleviates T-2 Toxin-Induced Testosterone Synthesis Disorder by Inhibiting ROS/p38/TORC2/CREB Axis
Source: Antioxidants (Basel). 2026 Aug 25;15(9):1060. doi: 10.3390/antiox15091060 (PMC13603086; doi:10.3390/antiox15091060)
Supplement: Supplementary file 1 [file antioxidants-15-01060-s001.zip › antioxidants-4448159-supplementary.pdf]

Table S1 Primer sequence and length of destination

Primer name	Gene accession mbers	Upstream and downstream primer sequence	Primer length (bp)	Product length (bp)
Mus β-actin	**NM_007393.5**	UP: 5' GATTACTGCTCTGGCTCCTA 3′	20	142
		LOW: 5' ATCGTACTCCTGCTTGCTGA 3′	20	
Mus ERK	**NM_001357115.1**	UP: CATGGAGACGGACCTTTACAAGC	23	164
		LOW: CACAAGTGGTGTTCAGCAGGAGG	23	
Mus JNK	**NM_001310453.2**	UP: TCCCAGCTGACTCAGAACATAACAA	25	84
		LOW: TGGACGCATCTATCACCAGCA	21	
Mus p38 MAPK	**NM_001357724.1**	UP: TCAGTCCATCATTCATGCGAAA	22	91
		LOW: AACGTCCAACAGACCAATCAC	21	
Mus 3β-HSD	**NM_001304800.1**	UP: 5' GTACATTTATGGGGAGAGAAGTCC 3′	24	130
		LOW: 5' CCAGGCCACATTGCCTACATA 3′	21	
Mus 17β-HSD	**NM_010475.2**	UP: 5' ACTTGGCTGTTCGCCTAGC 3′	19	117
		LOW: 5' GAGGGCATCCTTGAGTCCTG 3′	20	
Mus P450scc	**NM_001346787.1**	UP: 5' GGATGCGTCGATACTCTTCTCA 3′	22	146
		LOW: 5' GGACGATTCGGTCTTTCTTCCA 3′	22	
Mus P450c17	**NM_007809.3**	UP: 5' CGAGAAGTGCTGCGTATCAG 3'	20	125
		LOW: 5' AGTGCCCAGAGATTGACGAC 3'	20	
Mus StAR	**NM_011485.5**	UP: 5' AACGGGGACGAAGTGCTAAG 3′	20	184
		LOW: 5' CCGTGTCTTTTCCAATCCTCTG 3′	22	

Table S2 Antibodies required for Western Blot assay

Name of antibody	Host	Dilution	Manufacturer
Phospho-ERK1-T202 + ERK2-T185 Rabbit mAb	Rabbit	1:1500	ABclonal
ERK1/2 Rabbit mAb	Rabbit	1:1500	ABclonal
Phospho-JNK1/2/3-T183/T183-T221 Rabbit mAb	Rabbit	1:1500	ABclonal
JNK1/2/3 Rabbit mAb	Rabbit	1:1500	ABclonal
Phospho-p38 MAPK-T180 Rabbit pAb	Rabbit	1:1500	ABclonal
p38 MAPK Rabbit mAb	Rabbit	1:1500	ABclonal
HSD3B1 Rabbit pAb	Rabbit	1:800	ABclonal
HSD17B1 Rabbit pAb	Rabbit	1:1500	ABclonal
CYP11A1 Rabbit pAb	Rabbit	1:1500	ABclonal
CYP17A1 Rabbit mAb	Rabbit	1:1500	ABclonal
StAR Rabbit pAb	Rabbit	1:1500	ABclonal
phospho-TORC2 (Ser171) Rabbit pAb	Rabbit	1:1000	Bioss
Anti-TORC2 Rabbit pAb	Rabbit	1:800	Servicebio
Anti-Phospho-CREB (S133) Rabbit pAb	Rabbit	1:500	Servicebio
Anti-Lamin B1 Rabbit pAb	Rabbit	1:2000	Servicebio
Anti-beta Actin Mouse mAb	Mouse	1:1500	Servicebio
HRP Goat Anti-Rabbit IgG (H+L)	Rabbit	1:7000	ABclonal
HRP Goat Anti-Mouse IgG (H+L)	Mouse	1:5000	ABclonal

Figure S1 **LYC inhibited the activation of the p38-MAPK signaling pathway**

A

affinity (kcal/mol)	rmsd l.b.	rmsd u.b.
-7.3	0.000	0.000
-7.2	3.435	8.092
-7.1	31.833	35.054
-7.1	32.132	35.596
-7.1	5.932	17.342
-7.1	1.369	2.025
-6.9	31.612	33.676
-6.9	1.558	2.460
-6.8	31.935	35.314

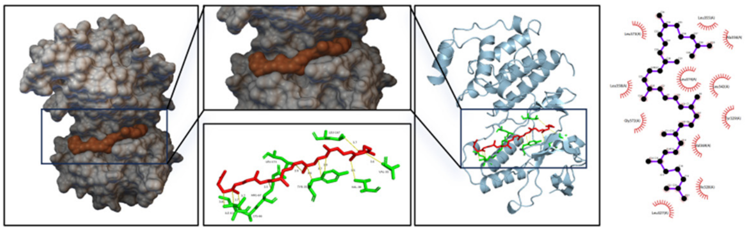

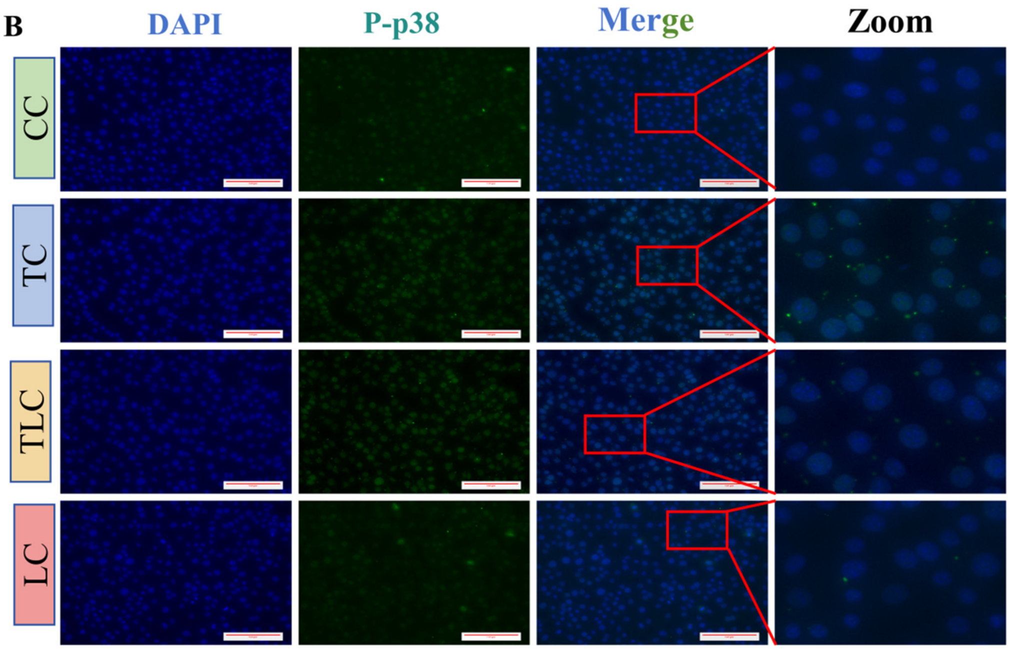

(A) Simulated molecular docking between LYC and p38; (B) TM3 cells P-p38 immunofluorescence.
